# Editorial

**DOI:** 10.1038/s41537-022-00262-8

**Published:** 2022-06-18

**Authors:** Robin Emsley

**Affiliations:** Department of Psychiatry, Faculty of Medicine and Health Sciences, Stellenbosch University, PO Box 241, Tygerberg, 8000 Cape Town, South Africa

## The way forward

This is my first editorial as Editor of *Schizophrenia*. I am honoured and delighted to be given this opportunity and I’m looking forward to working with the Schizophrenia International Research Society (SIRS) and with Springer Nature to make this a terrific journal. Many thanks to my predecessor, Jim Meador-Woodruff, for laying the foundations. He played an enormous role in establishing the journal and in defining its alignment with SIRS. I am currently discovering just how much of an effort he selflessly devoted to this task.

Little did we know, two and a half years ago, that our world was about change irrevocably. The World Health Organization declared the COVID-19 outbreak a Public Health Emergency of International Concern on 30 January 2020, and a pandemic on 11 March 2020. Since then, so much has happened that has affected us directly and indirectly. The pandemic has changed the way we work, and the way we communicate. Around the world we have all been subjected to various forms of lockdown and restrictions on our personal freedom, and many have suffered unthinkable personal losses. Both research and clinical settings were dramatically affected, and this has undoubtedly slowed progress in our understanding and management of individuals with schizophrenia. At the same time though, the pandemic has forced us to find new ways of functioning almost overnight. In- and outpatient services needed to adapt to ensure continuity of care while at the same time adhering to the covid protocols. Clinical research studies needed to be halted and protocols revised to comply with restrictions. Alternatives to existing educational and training practices needed to be found, and different ways of conducting scientific meetings evolved. We all dramatically upped our skills in virtual communication. While there is now a general feeling of cautious optimism that we’re emerging from the pandemic (after several false-starts!), experts warn that it’s not over yet. The virus has attenuated but remains highly prevalent and other post-infection complications are emerging. Nevertheless, we’re looking forward to returning to some measure of pre-pandemic life, with unrestricted travel and face-to-face congresses and networking opportunities. At the same time we have of necessity become proficient in conducting much of our business remotely. I anticipate that a good deal of this will be retained, given the huge savings in time and costs.

As the official SIRS journal, *Schizophrenia* will enthusiastically promote the SIRS mission which is to bring together researchers and clinicians in schizophrenia and related disorders. My vison is to continue to develop the journal and establish it as a leading resource in the field. I plan to broaden its scope and relevance and to extend its reach. To achieve this, the single highest priority is to ensure the consistent publication of articles of high quality that advance the knowledge base and to ensure that they are accessible to researchers and clinicians, as well as the broader community. To this end we will encourage submissions encompassing the entire spectrum of research into schizophrenia and related disorders, including preclinical, clinical, biological, pharmacological, psychological and sociological, as well as perspectives of persons with lived experience and carers. While there have been impressive advances in our understanding of the illness on many fronts, there remain so many unanswered questions. To effectively address these questions there is a great need for further developing cross-disciplinary collaborations and exploiting new technological advances. At the same time there is a need for more innovative thinking, and for stepping back from the data and challenging existing conceptual frameworks.

*Schizophrenia* will align itself closely with SIRS and will regularly inform readers of SIRS activities and initiatives. We will announce upcoming congresses, educational activities and grant and award opportunities. I will engage in ongoing discussion with the SIRS Executive Officers and Committees, and the journal will publish position statements and guidelines that have been developed by SIRS. A priority will be to encourage and facilitate the career development of young researchers.

While the core readership of the journal will comprise researchers in the field of schizophrenia, there is a much larger group of professional people who will benefit from the journal, namely those mental health workers who spend a great deal of their time managing patients with psychosis and their families. There is great interest amongst these clinicians to learn more about current best practices. To facilitate this, we will focus on bridging the gap between the often highly technical cutting-edge science in manuscripts and its clinical implications. We will solicit reviews and commentaries from researchers that provide clinical perspectives and highlight the relevance of research articles published in *Schizophrenia*.

Inclusivity, diversity and equity are core values of the journal. This relates particularly to gender, ethnicity and geographical representivity. I will endeavour to make the journal truly international, consistent with the stated aims of SIRS. While most of the published research is conducted in North America and Europe, there are excellent research initiatives in other parts of the world that often go by unnoticed. We hope to identify such research groups across the globe and to feature their work in the journal.

I’m appealing to researchers and readers to support our journal. Please consider submitting your best research to *Schizophrenia*. I am looking forward to working closely with the Associate Editors and other members of the Editorial Board. I will be relying on them for support and direction. I will do my best to provide a dedicated and committed service to individuals with schizophrenia, their families, carers and communities and to researchers and clinicians in the field.

